# Effect of plant growth regulators DA-6 and COS on drought tolerance of pineapple through bromelain and oxidative stress

**DOI:** 10.1186/s12870-023-04200-3

**Published:** 2023-04-05

**Authors:** XiaoKui Huang, GangShun Rao, XiaoDu Peng, YingBin Xue, HanQiao Hu, NaiJie Feng, DianFeng Zheng

**Affiliations:** 1 College of Coastal Agricultural Sciences, Guangdong Ocean University, Zhanjiang, 524000 Guangdong China; 2 Shenzhen Research Institute, Guangdong Ocean University, Shenzhen, 518000 Guangdong China

**Keywords:** Pineapple, Drought, Plant growth regulators, Bromelain, Oxidative stress

## Abstract

**Background:**

Due to global warming, drought climates frequently occur on land, and despite being drought resistant, pineapples are still subjected to varying degrees of drought stress. Plant growth regulators can regulate the stress tolerance of plants through hormonal effects. This experiment aims to investigate the regulatory effects of different plant growth regulators on Tainong- 16 and MD-2 Pineapple when subjected to drought stress.

**Results:**

In this experiment, we examined the regulatory effects of two different plant growth regulators, sprayed on two pineapple varieties: MD-2 Pineapple and Tainong-16. The main component of T1 was diethyl aminoethyl hexanoate (DA-6) and that of T2 is chitosan oligosaccharide (COS). An environment similar to a natural drought was simulated in the drought stress treatments. Then, pineapples at different periods were sampled and a series of indicators were measured. The experimental results showed that the drought treatments treated with T1 and T2 plant growth regulators had a decrease in malondialdehyde, an increase in bromelain and antioxidant enzyme indicators, and an increase in phenotypic and yield indicators.

**Conclusion:**

This experiment demonstrated that DA-6 and COS can enhance the drought resistance of pineapple plants to a certain extent through bromelain and oxidative stress. Therefore, DA-6 and COS have potential applications and this experiment lays the foundation for further research.

## Background

*Ananas comosus*, one of the most popular, edible tropical fruits and a member of the *Bromeliaceae* family, is grown in several tropical and subtropical countries, including Thailand, Indonesia, Malaysia, India, Kenya, China, and the Philippines [1]. For years, pineapple has been valued due to its pleasant and sweet taste as well as its wealth of nutrients such as fiber, numerous vitamins, manganese, and copper. Additionally, due to its low calorific value, pineapple has become a frequent component of diets among people who are concerned with their weight [1]. According to the report of the Food and Agriculture Organization of the United Nations, pineapples, avocados, and mangoes will remain the three most globally exported fruits in 2021, with pineapples as the most abundant commodity by far [2]. Since pineapples occupy an important market, it is vital to enhance their yield and ability to resist adversity.

Extreme and persistent weather can pose a threat to plants and limit crop yields. Among these, drought is a serious threat to plant growth and development, which will result in the over production of highly reactive oxygen species (ROS), such as singlet oxygen (^1^O_2_), hydrogen peroxide (H_2_O_2_), superoxide radical (O^2.-^), and the hydroxyl radical (·OH), causes oxidative damage to protein and lipids, which further leads to irreversible changes in protein structure and function, finally inhibit the photosynthetic process, growth, and yield [3]. However, exogenous application of PGRs in low concentrations often leads to significant improvements and high yields in fruit trees [4].And the strategies of exogenous plant growth regulators (PGRs) treatment have been utilized to effectively improve crop drought tolerance and save yield under drought stress [5]. Moreover, PGRs play critical regulatory functions in plant acclimatization to abiotic stress scenarios and could be handy to rectify the plant metabolism and development as well. Various abiotic stresses such as heat, salinity, drought, and cold stress regularly invigorate ABA build-up in plant tissues, which can then boost a variety of defense responses such as stomatal closure, metabolic changes, adjustments in plant growth and development [6]. When studying drought tolerance of crops, these related studies were also conducted, such as improving drought tolerance of maize with different regulators [7], studying putative roles of different PGRs in drought stress response [8], decreasing stress-induced damage by maintaining cell Membrane Structure, detoxifying ROS and regulating Antioxidant Systems [9], studying the role of exogenous hydrogen sulfide in improving drought tolerance in plants which occurred by inhibiting membrane damage、reducing the concentration of malondialdehyde, hydrogen peroxide, and increasing in gene expressions involved in ROS scavenging, cellular redox regulation, and proline biosynthesis genes [3].

Unlike animals, plants are unable to escape a harmful environment and instead must use their resources and energy to combat the stress when exposed to unfavorable conditions [10]. Pineapple is drought resistant [11]; however, frequent droughts continue to have an impact on pineapple and increase growth pressure. Countless hormonal signals finely regulate plant growth and development [12]. Hormone research is an important aspect of the research into the response of pineapple to biotic and abiotic stressors. While effective fertilization [13–16] and breeding works to combat stressors, the use of plant growth regulators (PGRs) can solve these problems faster and more efficiently [17].

Diethyl aminoethyl hexanoate (DA-6) is a novel artificial PGR and has been proven to ameliorate the effect of various abiotic stressors on critical physiological functions in many agricultural crops, a process that has been extensively applied crop production. In a recent study, DA-6 improves sunflower seed vigor under Al^3+^ stress by regulating Al^3+^ balance and ethylene metabolism [18]. DA-6 increases relay strip intercropping soybean grain by optimizing photosynthetic area and delaying leaf senescence [19]. DA-6 priming ameliorates seed germination via involvement in hormonal changes, osmotic adjustment, and dehydrin accumulation in white clover under drought stress [20]. DA-6 has positive effects on the growth and cadmium accumulation of tomato seedlings and can improve the physiology and selenium absorption of grape seedlings [21]. Exogenous DA-6 improves the low night temperature tolerance of tomatoes through regulation of cytokinin [22] and ameliorates low temperature stress by improving nitrogen metabolism in maize seedlings [23]. Furthermore, DA-6 regulates wheat grain filling after anthesis [24] and increases soybean pod setting and yield by regulating sucrose and starch content [25]. Specifically, DA-6 can increase the yield of soybean plants by improving their photosynthetic efficiency and increasing grain filling in the maize-soybean relay strip intercropping system [26]. Although numerous studied have described the beneficial effects of DA-6 on physiological and metabolic mechanisms of plants under unfavorable environmental conditions, no studies have been conducted to assess their effect on the ability of pineapples to resist drought.

Chitin oligosaccharides (CTOs) and their related compounds chitosan oligosaccharides (CSOs) are collectively known as chitooligosaccharides (COSs) [27]. COSs, the oligomers of chitosan, are a non-toxic, efficient, novel antioxidants that act as signal molecules as well as chemical agents against abiotic stress [28]. In recent studies, COSs have been shown to have a certain degree of antifungal and antioxidant properties as well [29–32]. Specifically, COSs have been found to improve the growth and photosynthetic characteristics of wheat seedlings [33] and have a positive effect on the thermotolerance of arabidopsis [34]. COSs enhance vindoline and catharanthine accumulation, antioxidant enzyme activity, and gene expression levels in *Catharanthus roseus* leaves [35]. COSs enhance the production of phenolic compounds during barley germination to improve the antioxidant capacity of malt [36]. COSs alleviate oxidative stress generated in tomato plants under stress by UV-B radiation [37].

Bromelain is a collective name for proteolytic enzymes or proteases and describes a crude extract of pineapples with endopeptidase activity [38] that can be taken from tissues, including the stems, fruits, and leaves, of the pineapple and other plant species of the family *Bromeliaceae* [39]. Bromelain has several industrial and biomedical applications and has been used in the food, baking, leather, textile, and cosmetic industries [40]. Bromelain is involved in the development, protein turnover, and degradation of damaged proteins in response to various abiotic and biotic stressors [41]. Additionally, it contributes, to a certain extent, to the antioxidant capacity of pineapple [42]. In this experiment, we use bromelain as a metric to measure the resistance of pineapple to drought.

In recent years, a series of studies examined the high yield and resilience to adversity of pineapple, including various studies on flowering [43–46], reduction of browning [47–50], quality and harvest [51–54], growth and ripening changes [55], salt tolerance [56], tissue culture and breeding [57–60], growth of seedlings [61], irrigation of cultivation [62], the effects of drought on growth [39], and the effects of high temperatures postharvest [63]. Despite pineapples being drought-tolerant, extreme and persistent drought exert negative impacts on yield, requiring attention to be paid to this issue. PGRs are employed in agriculture, horticulture, and viticulture to decrease susceptibility to biotic and abiotic stressors, improve morphological structure, facilitate harvesting, improve quantitative and qualitative increases in yield, and modify plant constituents [17]. Researchers combine different plant hormones to be used as PGRs to enhance the resistance of pineapple to adversity more effectively; however, in reality, more efficient PGRs are necessary to cope with extreme changes in environment. The aim of this study is to enhance the resistance of pineapple to drought through PGRs.

Thus, we treated two varieties of pineapple, Tainong-16 and MD-2 Pineapple, with two PGRs, DA-6 and COS, to measure changes in a various indexes, such as bromelain and those of oxidative stress, upon exposure to drought-like conditions. This study provides new options for enhancing the resistance of pineapple to drought and sets a foundation for more extensive study of the resistance mechanisms.

## Results

### Effects on soil volumetric water content under natural drought conditions

**Fig. 1:**
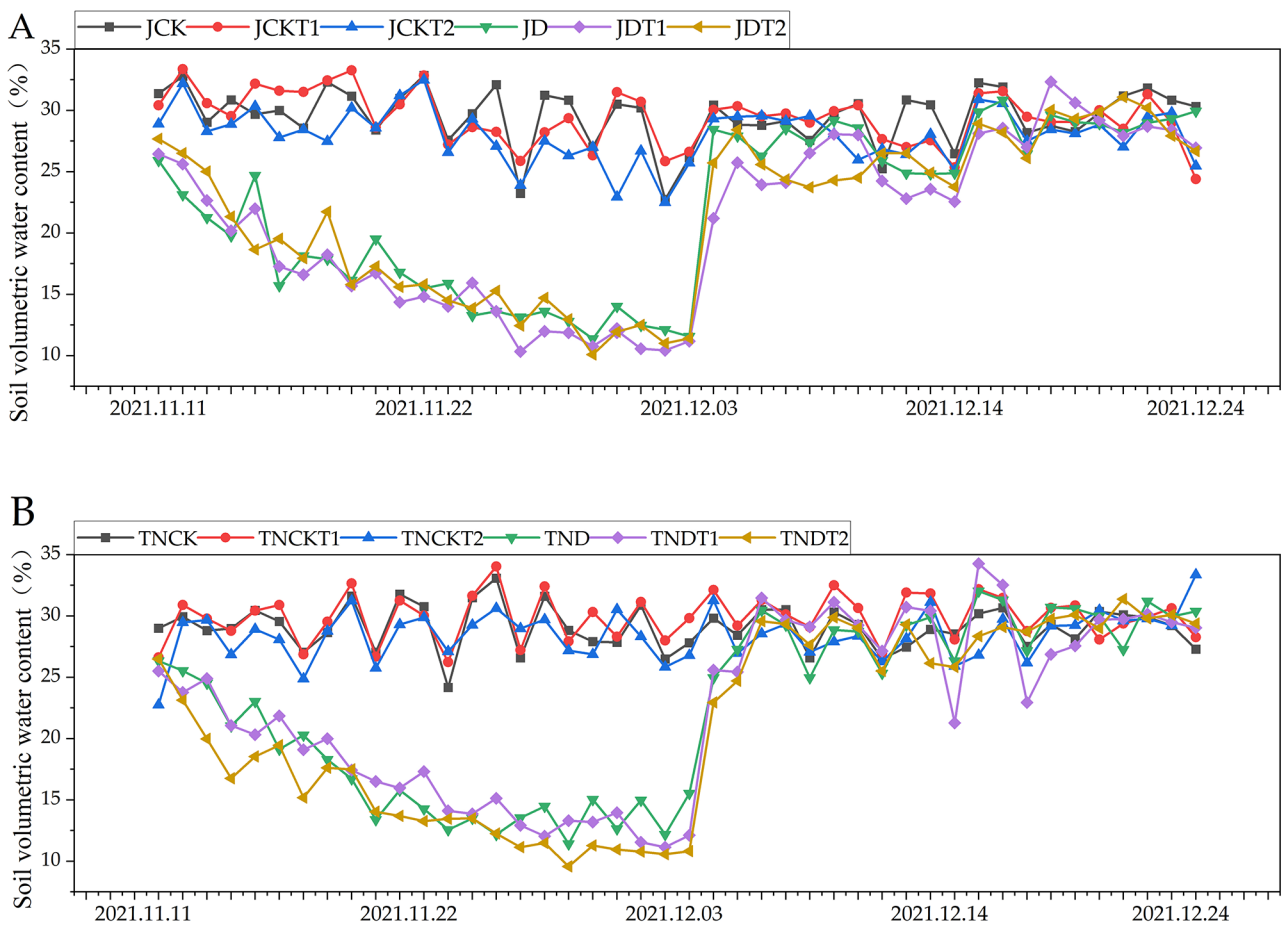
The soil volumetric water content during the five periods in MD-2 Pineapple **(A)** and Tainong-16 **(B)** treatments

In Fig. 1, due to continuous irrigation, the soil volumetric water content of the normal watering treatments of both MD-2 Pineapple and Tainong-16 continuously exceeded 25%, while that of the drought treatments declined to approximately 10%. With the restoration of irrigation, the volume moisture content of the soil in the drought group recovered to more than 25%.The soil conditions were considered a “mild drought” when the soil volumetric water content reached 15%–20% and a “severe drought” when it reached 10–15%.

### Effect of DA-6 and COS on bromelain activity in natural drought conditions

**Fig. 2:**
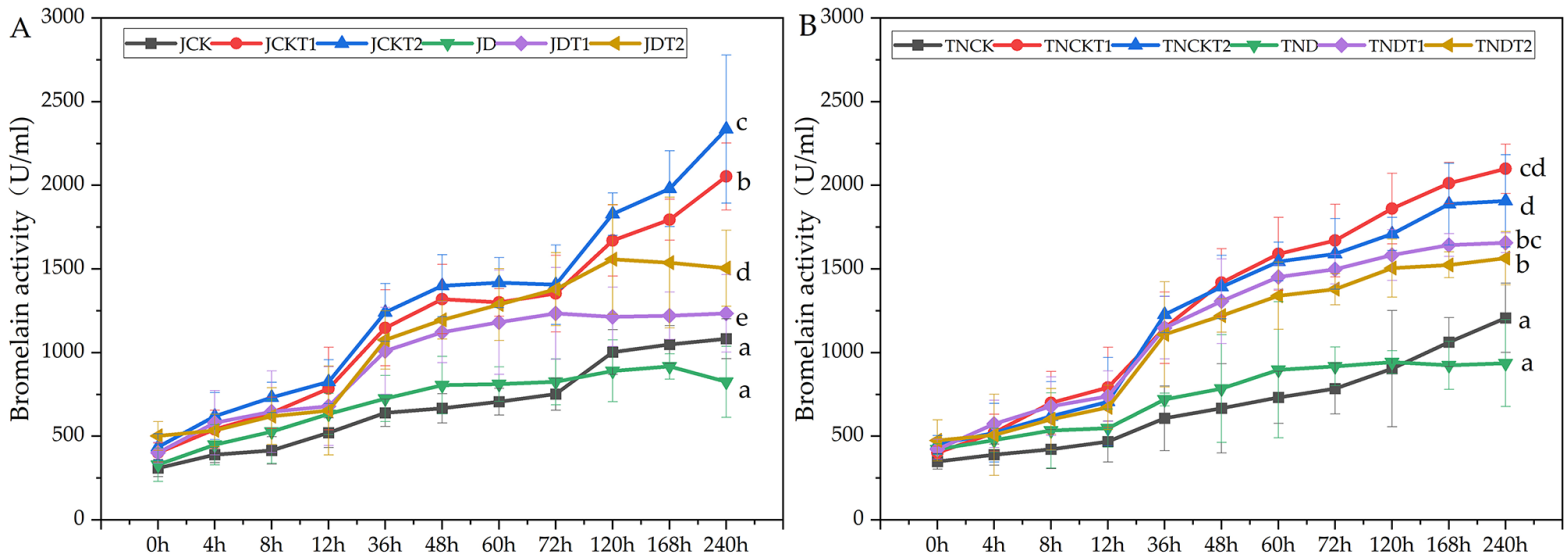
The activities of bromelain during the eleventh periods in MD-2 Pineapple **(A)** and Tainong-16 **(B)** treatments. Values are presented as the mean ± SE (n = 4) of four biological replicates. Lower case letters above columns indicate significant difference (p<0.05)among treatments

In the MD-2 Pineapple treatments shown in Fig. 2, analysis with a two-way repeated measures ANOVA, the treatments JD, JDT1, and JDT2 have significant differences in protease activity from each other. Specifically, that of JDT1 increased by 49.6% and that of JDT2 increased by 82.4% compared to that of JD. At 120 h, the trends in drought treatments tended to decrease.

In the Tainong-16 treatments shown in Fig. 2, using a two-way repeated measures ANOVA, we found that the TND had significant differences in protease content from the TNDT1 and TNDT2 treatments. Specifically, that of TNDT1 increased by 76.8% and that of TNDT2 increased by 66.9% compared to TND.

### Effect of DA-6 and COS on superoxide dismutase (SOD) activity in natural drought conditions

**Fig. 3:**
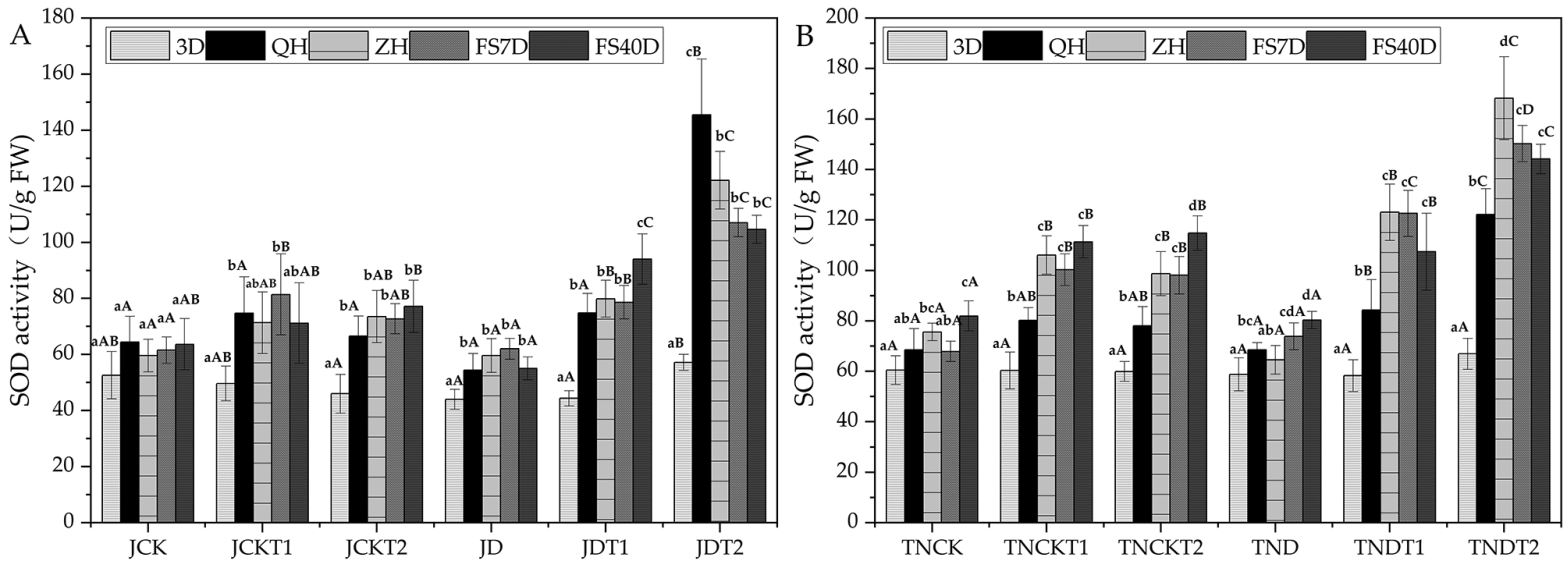
The activities of SOD in MD-2 Pineapple **(A)** and Tainong-16 **(B)** treatments between different periods and different treatments. Values are presented as the mean ± SE (n = 4) of four biological replicates. Lower case letters above columns indicate significant difference (p<0.05)within treatments. Capital letters above columns indicate significant difference (p<0.05) among treatments

In Fig. 3, using a one-way ANOVA within treatments, the treatments of MD-2 Pineapple JDT1 and JDT2 at sampling time 3D showed significantly different SOD activity from other sampling times, except JD. In the treatments of Tainong-16 TNDT1 and TNDT2, the SOD activity at sampling time 3D were significantly different from other periods in their treatments, while that of TND at sampling time 3D was significantly different from that at QH but not ZH. Thus, it can be determined that significant differences shown at sampling time ZH.

On the other hand, using one-way ANOVA between treatments, in the treatments of MD-2 Pineapple, the SOD activity of JD was significantly different from that of JDT1 and JDT2 at both QH and ZH. At the sampling time QH, the SOD of JDT1 had increased by 37.6% and that of JDT2 had increased by 167.2% compared to JD. At the sampling time ZH, the SOD of JDT1 had increased by 33.9% and that of JDT2 had increased by 104.8% compared to JD. In the treatments of Tainong-16, the SOD activity of TND was significantly different from that of TNDT1 and TNDT2 at both QH and ZH as well. At sampling time QH, the SOD activity of TNDT1 had increased by 23% and that of TNDT2 had increased by 78.3% compared to TND. At sampling time ZH, the SOD activity of TNDT1 had increased by 90.5% and that of TNDT2 had increased by 160.4% compared to TND.

### Effect of DA-6 and COS on peroxidase (POD) in natural drought conditions

**Fig. 4:**
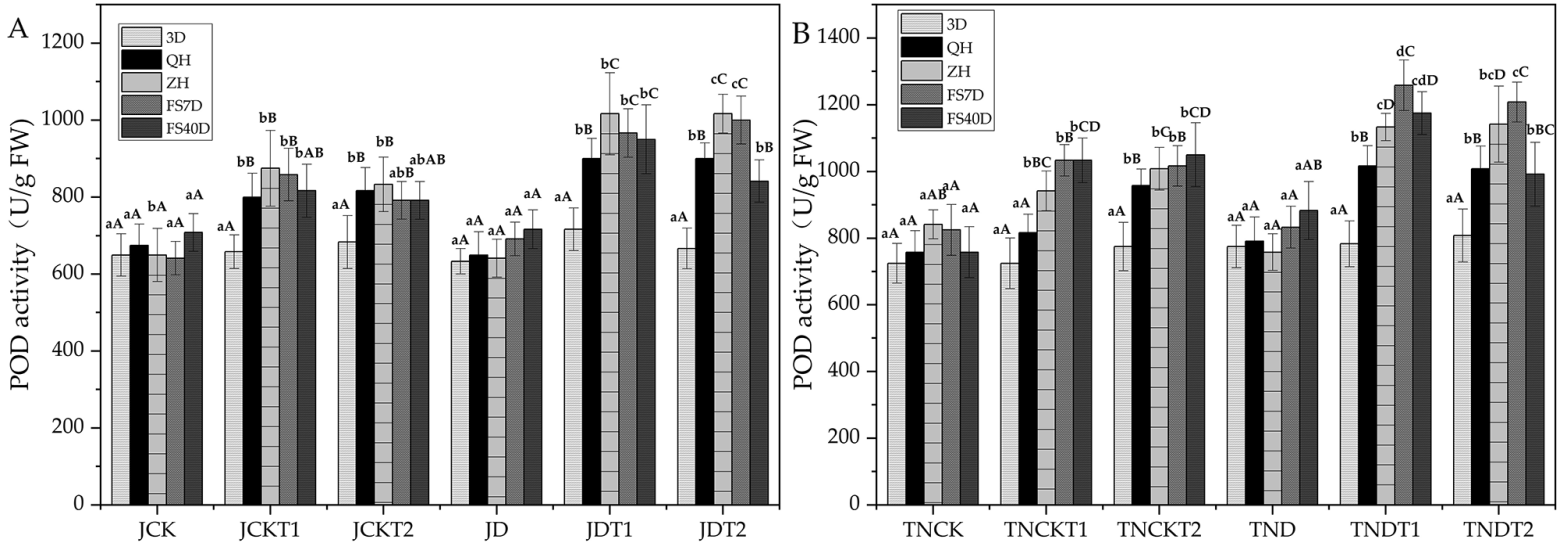
The activities of POD in MD-2 Pineapple **(A)** and Tainong-16 **(B)** treatments between different periods and different treatments. Values are presented as the mean ± SE (n = 4) of four biological replicates. Lower case letters above columns indicate significant difference (p<0.05)within treatments. Capital letters above columns indicate significant difference (p<0.05) among treatments

In Fig. 4, using one-way ANOVA within treatments, in the treatments of MD-2 Pineapple JDT1 and JDT2, the POD activity at sampling time 3D were significantly different from those of other periods in their treatments, except for JD. In the treatments of Tainong-16 TNDT1 and TNDT2, the POD activity at sampling time 3D were significantly different from those of other periods in their treatments except for TND.

On the other hand, using one-way ANOVA between treatments, in the treatments of MD-2 Pineapple, the POD activity of JD was significantly different from that of JDT1 and JDT2 at both QH and ZH. At sampling time QH, the POD activity of JDT1 had increased by 38.5% and that of JDT2 had increased by 38.5% compared to JD. At sampling time ZH, the POD activity of JDT1 had increased by 58.4% and that of JDT2 had increased by 58.4% compared to JD. In the treatments of Tainong-16, the POD activity of TND was significantly different from that of TNDT1 and TNDT2 at both QH and ZH. At sampling time QH, the POD activity of TNDT1 had increased by 28.4% and that of TNDT2 had increased by 27.3% compared to TND. At sampling time ZH, the POD of TNDT1 had increased by 49.4% and that of TNDT2 had increased by 50.5% compared to TND.

### Effect of DA-6 and COS on catalase (CAT) in natural drought conditions

**Fig. 5:**
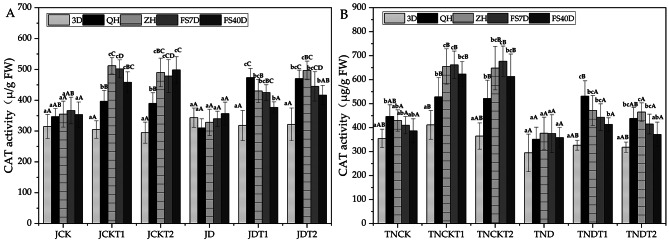
The activities of CAT in MD-2 Pineapple **(A)** and Tainong-16 **(B)** treatments between different periods and different treatments. Values are presented as the mean ± SE (n = 4) of four biological replicates. Lower case letters above columns indicate significant difference (p<0.05)within treatments. Capital letters above columns indicate significant difference (p<0.05) among treatments

In Fig. 5, using one-way ANOVA within treatments, in the treatments of MD-2 Pineapple JDT1 and JDT2, the CAT activity at sampling time 3D were significantly different from that of other periods in their treatments except for JD. In the treatments of Tainong-16 TNDT1 and TNDT2, the CAT activity at sampling time 3D were significantly different from that of other periods in their treatments except for TND.

On the other hand, using one-way ANOVA between treatments, in the treatments of MD-2 Pineapple, the CAT activity of JD was significantly different from that of JDT1 at QH and those of JDT1 and JDT2 at ZH. At sampling time QH, the CAT activity of JDT1 had increased by 52.7% and that of JDT2 had increased by 51.6% compared to JD. At sampling time ZH, the CAT activity of JDT1 had increased by 31% and that of JDT2 had increased by 51.3% compared to JD. In the treatments of Tainong-16, that CAT activity of TND was significantly different from those of TNDT1 and TNDT2 at QH and ZH. At sampling time QH, the CAT activity of TNDT1 had increased by 51.2% and that of TNDT2 has increased by 24.6% compared to TND. At sampling time ZH, the CAT activity of TNDT1 had increased by 25.2% and that of TNDT2 had increased by 23.4% compared to TND.

**Fig. 6:**
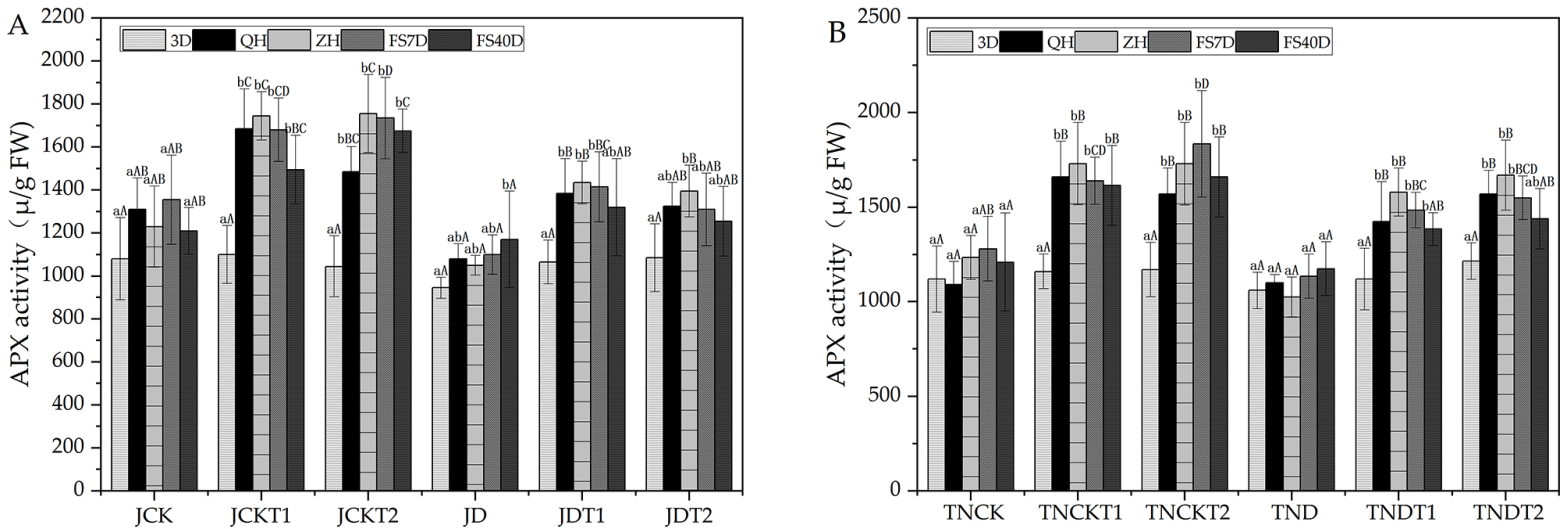
The activities of APX in MD-2 Pineapple **(A)** and Tainong-16 **(B)** treatments between different periods and different treatments. Values are presented as the mean ± SE (n = 4) of four biological replicates. Lower case letters above columns indicate significant difference (p<0.05)within treatments. Capital letters above columns indicate significant difference (p<0.05) among treatments

In Fig. 6, using one-way ANOVA within treatments, in the treatments of MD-2 Pineapple, the APX activity in JDT1 at sampling time 3D was significantly different from that at both QH and ZH in its treatments. Additionally, the APX activity at sampling time 3D of JDT2 was significantly different from that at ZH but not JD. In the treatments of Tainong-16 TNDT1 and TNDT2, the APX activity at sampling time 3D were significantly different from those at QH and ZH in their respective treatments.

On the other hand, using one-way ANOVA between treatments, in the treatments of MD-2 Pineapple JDT1 and JDT2, that APX activity at sampling time 3D were significantly different from those of ZH in their respective treatments. At sampling time QH, that APX activity of JDT1 had increased by 28% and that of JDT2 had increased by 23% compared to JD. At sampling time ZH, the APX activity of JDT1 had increased by 36.7% and that of JDT2 had increased by 32.8% compared to JD. In the treatments of Tainong-16, the APX activity of TND was significantly different from those of TNDT1 and TNDT2 at both QH and ZH. At sampling time QH, the APX activity of TNDT1 had increased by 30% and that of TNDT2 had increased by 43% compared to that of TND. At sampling time ZH, the APX activity of TNDT1 had increased by 54.1% and that of TNDT2 had increased by 62.9% compared to that of TND.

### Effect of DA-6 and COS on malondialdehyde (MDA) in natural drought conditions

**Fig. 7:**
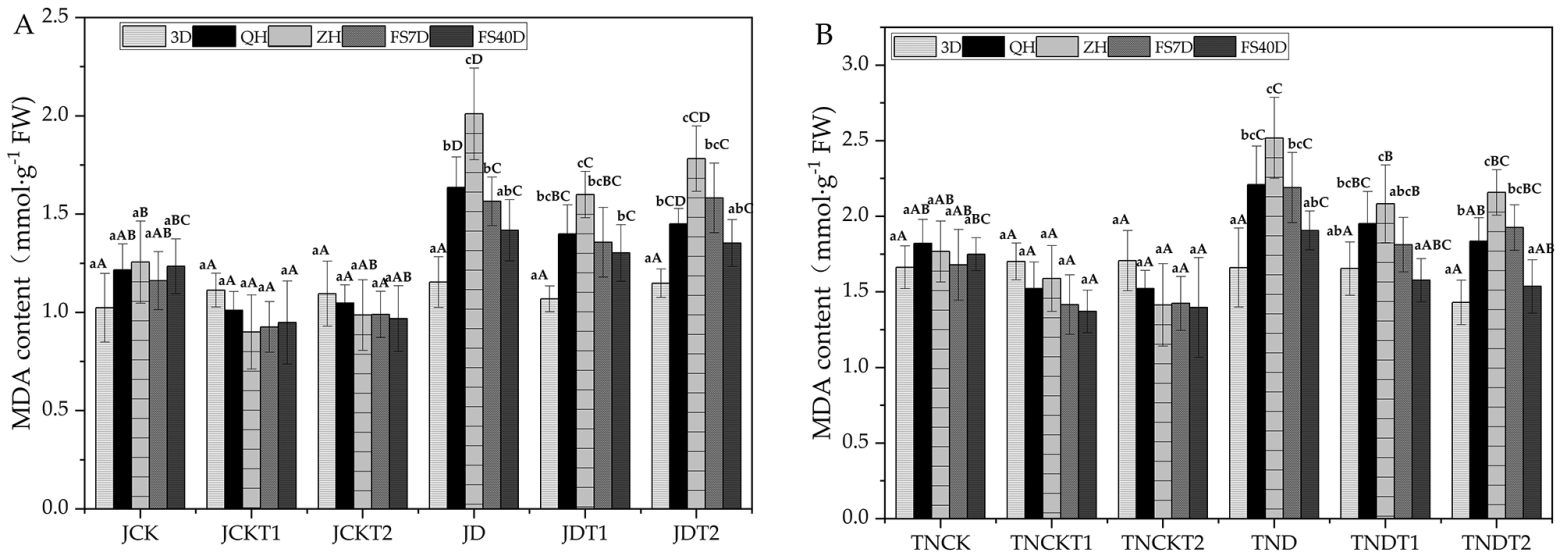
The content of MDA in MD-2 Pineapple **(A)** and Tainong-16 **(B)** treatments between different periods and different treatments. Values are presented as the mean ± SE (n = 4) of four biological replicates. Lower case letters above columns indicate significant difference (p<0.05)within treatments. Capital letters above columns indicate significant difference (p<0.05) among treatments

In Fig. 7, using one-way ANOVA within treatments, in the treatments of MD-2 Pineapple JD, JDT1, and JDT2, the MDA contents at sampling time 3D were significantly different from those at both QH and ZH in their respective treatments except for the normal watering treatments. In the treatments of Tainong-16, the MDA content at sampling time 3D of TNDT1 was significantly different from that at ZH in its treatments and those at 3D of TND and TNDT2 were significantly different from those at QH and ZH in their respective treatments except for the normal watering treatments.

On the other hand, using one-way ANOVA between treatments, in the treatments of MD-2 Pineapple, the MDA content of JD was significantly different from that of JDT1 at both QH and ZH. At sampling time QH, the MDA content of JDT1 had decreased by 14.4% and that of JDT2 had decreased by 11.3% compared to that of JD. At sampling time ZH, the MDA content of JDT1 had decreased by 20.4% and that of JDT2 had decreased by 11.3% compared to that of JD. In the treatments of Tainong-16, the MDA content of TND was significantly different from those of TNDT2 at QH and TNDT1 at ZH. At sampling time QH, the MDA content of TNDT1 had decreased by 11.7% and that of TNDT2 had decreased by 17% compared to that of TND. At sampling time ZH, the MDA content of TNDT1 had decreased by 17.3% and that of TNDT2 had decreased by 14.3% compared to that of TND.

### Effect of DA-6 and COS on leaf phenotypes in natural drought conditions

**Fig. 8:**
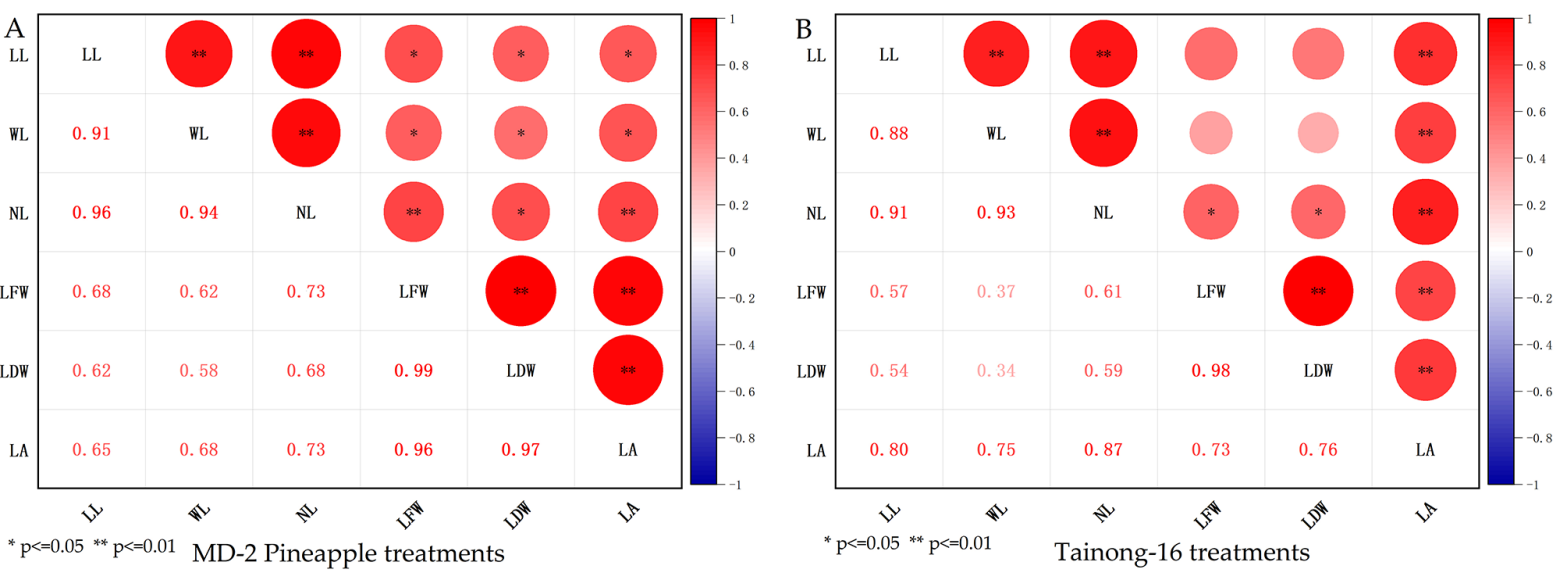
The correlations between the leaf length of the longest leaf (LL), the leaf width of the longest leaf (WL), the leaf number (NL), the leaf fresh weight (LFW), the leaf dry weight (LDW), the leaf area (LA) in MD-2 Pineapple **(A)** and Tainong-16 **(B)** treatments. * indicates significant correlation at 0.05 level; ** indicates significant correlation at 0.01 level

In Fig. 8, using Pearson correlation analyses, in the treatments of MD-2 Pineapple, the indicators of longest leaf (LL), leaf width (WL), leaf number (NL), leaf fresh weight (LFW), leaf dry weight (LDW), and leaf area (LA) all have a significant correlation. In the treatments of Tainong-16, apart from the correlations between indicators LL and WL and LFW and LDW, the rest of the indicators have a significant correlation.

### Effect of DA-6 and COS on pineapple friut in natural drought conditions

**Fig. 9:**
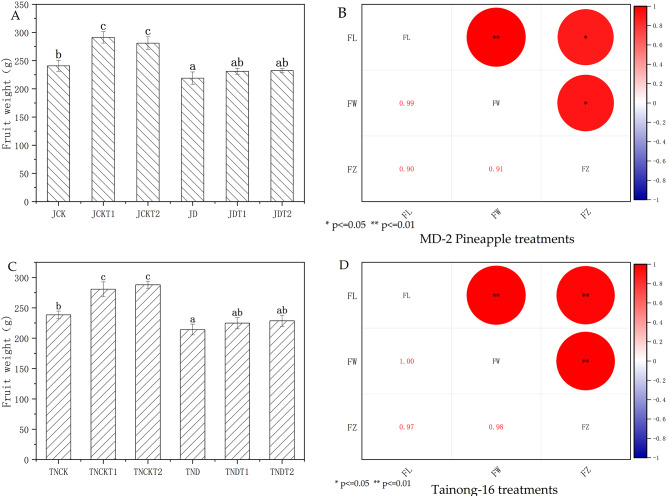
The content of fruit weight in MD-2 Pineapple **(A)** and Tainong-16 **(C)** treatments. The correlations between the fruit length (FL), the fruit width (FW), the fruit weight (FZ) in MD-2 Pineapple **(B)** and Tainong-16 **(D)** treatments. Values are presented as the mean ± SE (n = 4) of four biological replicates. * indicates significant correlation at 0.05 level; ** indicates significant correlation at 0.01 level

In Fig. 9, using Pearson correlation analyses in the treatments of MD-2 Pineapple, the indicators of fruit length (FL), fruit width (FW), and fruit weight (FZ) have a significant correlation. In the treatments of Tainong-16, the indicators of FL, FW, and FZ also have a significant correlation.

On the other hand, using one-way ANOVA between treatments, in the treatments of MD-2 Pineapple, the yield of JCK was not significantly different from those of JDT1 and JDT2, yet was significantly different from that of JD. The yield of JD had decreased by 9.1% compared to that of JCK, that of JDT1 had decreased by 4% compared to that of JD, and that of JDT2 had decreased by 3.6% compared to that of JD. In the treatments of Tainong-16, the yield of TNCK was not significantly different from those of TNDT1 and TNDT2, yet was significantly different from that of TND. The yield of TND had decreased by 10.1% compared to that of TNCK, that of TNDT1 had decreased by 5.7% compared to that of TND, and that of TNDT2 had decreased by 4.1% compared to that of TND.

## Discussion

In plants, drought stress is usually manifested by growth retardation, yellowing and photosynthesis inhibition of roots and leaves [64]. In this study, drought stress severely inhibited the growth of pineapple, which may be caused by changes in the physiological and biochemical level of cellular processes in pineapple. However, exogenous use of DA-6 and COS restored these growth parameters.

Exposure to drought stress affects cellular processes, affects the mechanisms of photosynthesis, and causes the accumulation of reactive oxygen species to exceed the ability of the plant itself to remove them [5]. In this study, pineapples exposed to drought stress produced significantly more oxidative stress than controls. On the contrary, the MDA content of MD-2 and Tainong-16 under drought stress was decreased after spraying with DA-6 and COS, indicating the important role of DA-6 and COS in alleviating oxidative damage.

From the perspective of bromelain activity, in this study, compared with the control, the pineapples subjected to drought stress inhibited the increase of bromelain activity, while on the contrary, the spray of DA-6 and COS promoted the increase of bromelain activity of MD-2 and Tainong-16, indicating that DA-6 and COS promoted the production of antioxidant enzymes in pineapple in response to oxidative stress.

From the perspective of phenotypic correlation of pineapple leaves, in this study, there was a positive correlation between the leaf indexes of pineapple leaves subjected to drought stress and those after rehydration. It can be seen from the figure that the spraying of DA-6 and COS promoted the overall phenotypic growth of MD-2 and Tainong-16 leaves.

From the perspective of pineapple fruit weight, in this study, compared with the control group, pineapples subjected to drought stress inhibited the growth of pineapple fruit weight, while on the contrary, the spray of DA-6 and COS alleviated the reduction of MD-2 and Tainong-16 pineapple fruits, and there was no significant difference in weight compared with the normal treatment group. From the correlation of pineapple fruit indexes, the spraying of DA-6 and COS promoted the overall growth of MD-2 and Tainong-16 fruit indexes.

In this experiment, after spraying the pineapple plants with the PGRs DA-6 and COS, we measured indexes concerning ROS damage to lipids, enzymatic antioxidants, non-enzymatic antioxidants, phenotypic indicators, and yield to observe the resistance to drought conditions. First, the decline of MDA content signifies lipid damage, potentially because DA-6 and COS positively regulated within pineapple to relieve damage. According to the data, COS more effectively regulate than DA-6. Second, the increase in enzymatic antioxidants, such as SOD, POD, CAT, and APX, counteracts oxidative stress in pineapple, potentially because DA-6 and COS positively regulated these enzymes. Third, the positive correlations between leaf indicators may be due to DA-6 and COS positively regulating leaf growth in pineapple. Finally, the reduced fruit weight loss may be due to DA-6 and COS helping pineapple growth when faced with drought stress to mitigate the loss of pineapple weight.

Overall, we suggest that DA-6 and COS are involved in the regulation of pineapple to increase its ability to resist drought through indexes of oxidative stress and increasing the amount of bromelain. Due to the regulation of antioxidant systems and the increasing of bromelain, pineapple plants counteract the damage to protect cells from free radicals according to indexes.

From domestic and international research, there are relative research we can learn methods and technologies for use in further experiments. For example, validating the genes related to resistance of pineapple in Arabidopsis [65, 66], studying the molecular mechanisms of resistance in pineapple through transcriptomic and metabolomic analyses [67–70], analyzing resistance mechanisms through the expression of genes [71], studying the regulation of resistance of Arabidopsis thaliana by pineapple-related kinase gene [11, 65], studying the regulation ability of beneficial elements on plant tolerance to abiotic stress [72], studying the effect of combining lipoic acid and melatonin against oxidative stress [73], studying the effect of melatonin against abiotic stress [74], studying the ability of other PGRs to alleviate drought stress in rice.

In this experiment, according to the analysis of the indexes of bromelain and oxidative stress, we conclude that improvement of pineapple yield and drought resistance can be applied not only to breeding and fertilizers, but also to the use of PGRs in effectively improving the resistance of pineapple. For the further research, we can elucidate the regulatory process of pineapple in the way of gene expression. Moreover, we can analyze the related genes and metabolic pathways according to the up-regulation or down-regulation of genes by transcriptome sequencing. Additionally, we can select genes that significantly change for further research, including bioinformatics analysis, genes expression analysis, genes functional validation, and gene interactions. Finally, we can simulate real drought conditions to transfer pineapple to a field during the experiment. In conclusion, we regulated the resistance of pineapple to drought through DA-6 and COS and found that PGRs have the ability to regulate pineapple through the measured indexes such as bromelain and oxidative stress.

## Conclusions

In this experiment, we spray treated MD-2 pineapple and Tainong-16 with two PGRs, DA-6 and COS, and simulated natural drought conditions. Then, sampling was performed at designated sampling times to measuring indexes such as lipid damage, enzymatic antioxidants, phenotypic indicators, and yield. We found that several indexes such as bromelain, antioxidant enzymes showed improved performance in resistance of MD-2 pineapple and Tainong-16 to drought, and reduced the loss of pineapple weight. The results showed that DA-6 and COS can regulate in the antioxidant system involved in drought resistance through bromelain and oxidative stress, counteracting the damage from ROS. This research supplements the lack of previous studies on the regulation of DA-6 and COS in the drought response of pineapple. Further research on the mechanisms involved in the drought resistance of pineapple is necessary. For more extensive study, our results need to be validated by transcriptomics and metabolomics in the future.

## Methods

### Plant material

This experiment used two different varieties of pineapple, Tainong-16 and MD-2 Pineapple, provided by the South Subtropical Crop Research Institute to act as the experiment material. First, 104 plants of each variety were processed by disinfection and drying. Then, the plants were planted individually inside a glass greenhouse located at the Institute of Agricultural Research, Guangdong Ocean University. The two PGRs were provided by the Chemical Control Center of Guangdong Ocean University.

### Media and treatments

This experiment used 208 plastic pots, each were 24 cm in height and had an inner diameter of 26 cm. Each pot was filled with 12 kg of lateritic soil mixed with 500 g organic fertilizer, 5.1 g urea, 16.3 g calcium superphosphate, and 4.7 g potassium sulfate. The Chemical Control Center of Guangdong Ocean University prepared the two PGRs, labelled T1 and T2. The main component of T1 is DA-6 at a concentration of 40 mg/L and that of T2 is COS at a concentration of 30 mg/L. DA-6 and COS were sprayed when MD-2 Pineapple and Tainong-16 Pineapple are normal growing. On the basis of a spray volume of 30 L per acre, the T1 treatment and T2 treatments were sprayed with a total of 172 ml T1 and T2, respectively, while the normal watering and drought treatments were sprayed with 172 ml distilled water. This experiment divided each pineapple variety into 6 treatments, each of which contained 18 plants. To distinguish between the experimental treatments, the abbreviations are as follows for MD-2 Pineapple and Tainong-16: JCK and TNCK stand for the normal watering treatments, JCKT1 and TNCKT1 stand for the normal watering with T1 spray treatments, JCKT2 and TNCKT2 stand for the normal watering with T2 spray treatments, JD and TND stand for the drought treatments, JDT1 and TNDT1 stand for the drought with T1 spray treatments, and JDT2 and TNDT2 stand for the drought with T2 spray treatments, respectively (Table 1).

This experiment used drippers to control the plant watering. When all treatments were sprayed with PGRs, and the first sampling was taken three days later, the drought treatments proceeded to natural drought. To simulate natural drought conditions in drought treatments, the dripper was pulled out to stop watering. At the same time, normal watering was maintained in the other treatments. After rehydration, all treatments kept normal watering.

**Table 1:** The table indicates the abbreviations of the treatments used in the article

diethyl aminoethyl hexanoate (DA-6) Chitosan oligosaccharide (COS)	T1 T2
MD-2 Pineapple Tainong-16	J TN
Normal watering treatments of MD-2 pineapple Normal watering and T1 spray treatments of MD-2 pineapple Normal watering and T2 spray treatments of MD-2 pineapple Drought treatments of MD-2 pineapple Drought and T1 spray treatments of MD-2 pineapple Drought and T2 spray treatments of MD-2 pineapple Normal watering treatments of Tainong-16 Normal watering and T1 spray treatments of Tainong-16 Normal watering and T2 spray treatments of Tainong-16 Drought treatments of Tainong-16 Drought and T1 spray treatments of Tainong-16 Drought and T2 spray treatments of Tainong-16	JCK JCKT1 JCKT2 JD JDT1 JDT2 TNCK TNCKT1 TNCKT2 TND TNDT1 TNDT2

### Sampling times and determination of soil volume water content

For the measurement, five sampling times were delineated as follows: 1) three days after spraying (3D), 2) the period of mild drought in which soil volume water content was 15–20% (QH), 3) the period of severe drought in which soil volume water content was 10%–15% (ZH), 4) seven days after rehydration (FS7D), and 5) forty days after rehydration (FS40D) (Table 2). The volume moisture content of soil is measured by Soil temperature and humidity detector (TR-6D, SHUNKEDA, Beijing, China). At each time of sampling, we sampled the tip of the leaf and saved in liquid nitrogen. For the measurement of bromelain, sampling was performed eleven times, at 0 h, 4 h, 8 h, 12 h, 36 h, 48 h, 60 h, 72 h, 120 h, 168 h, and 240 h after spraying with PGRs. For the measurements of leaf phenotype, we chose two times at which to sample, severe drought and twenty days after rehydration. For the measurement of indicators concerning the pineapple fruits, we sampled the fruit without stalk when they were ripe.

**Table 2:** The table indicates the abbreviations of the sampling periods used in the article

Three days after spraying Mild drought Severe drought Seven days after rehydration Forty days after rehydration	3D QH ZH FS7D FS40D

### Determination of lipid peroxidation

Lipid peroxidation was indicated by the content of malondialdehyde (MDA), which was measured using thiobarbituric acid (TBA) [40]. Briefly, 0.1 g leaves were extracted with 10 mL of 10% trichloroacetic acid (TCA) and then centrifuged at 10,000×g for 20 min. The equal volume of TBA was added to the supernatant and incubated at 95°C for 30 min, and then it was cooled on ice immediately. The absorbances at 450, 532, and 600 nm were measured using an ultraviolet spectrophotometer. (UV-2100, UNICO, Shanghai, China) after centrifugation 20 min at 10,000×g.

### Determination of bromelain and antioxidant enzymes

The bromelain activity assay was based on estimation of amount of small molecular weight digestion products (Trichloroacetic acid (TCA) soluble material) formed from proteins due to proteolytic action of the enzyme [75]. Proteolytic activity of bromelain was measured. The assay consisted of 5 ml solution of 0.75% casein prepared in anhydrous disodium phosphate buffer (50 mM, pH 7). The pH was adjusted using slow addition of 0.1 N HCl. The solution was brought to 37°C by pre-incubation for 10 min. 0.1 g leaves were extracted with 10 mL of 10% trichloroacetic acid (TCA) and then centrifuged at 10,000 × g for 20 min. To this substrate, a known volume of enzyme was added after diluting it to 1 ml with activating buffer. The 30 mM cysteine hydrochloride monohydrate in 6 mM disodium EDTA was used as an activating buffer. Casein proteolysis was stopped after 10 min by addition of 5 mL of 30% (w/v) TCA and was allowed to stand for 30 min at 37°C. Thereafter, the solution was cooled to room temperature and filtered twice using Whatman filter paper (No. 42). Absorbance of the filtrate was measured at 280 nm using UV-Vis spectrophotometer against the tyrosine standard plot of absorbance versus tyrosine concentration (µg mL^-1^). One unit of BML was taken as the amount of enzyme which while acting on the casein substrate under specified conditions, produces one microgram of tyrosine per minute.

The superoxide dismutase (SOD) activity in leaves was determined by the rate of inhibition of nitro blue tetrazolium (NBT) reduction at 560 nm, with xanthine oxidase as a H_2_O_2_ producing agent, as previously described [76]. The amount of enzyme required for 50% inhibition of the photochemical reduction of NBT was defined as one unit of SOD activity. 0.1 g leaves were extracted with 10 mL of 10% trichloroacetic acid (TCA) and then centrifuged at 10,000 × g for 20 min.

POD activity was determined from increased absorbance at 470 nm for 3 min [77]. POD activity was calculated using the extinction coefficient of guaiacol (26.6 mM^− 1^ cm^− 1^) and a complete reaction mixture without H_2_O_2_ was used as a blank. 0.1 g leaves were extracted with 10 mL of 10% trichloroacetic acid (TCA) and then centrifuged at 10,000 × g for 20 min.

Catalase (CAT) activity was measured by the method proposed by M. Ekincia, S. Orsb et al. [78]. In brief, the total volume of 3.0 mL reaction solution comprising 1.5 mL of 50 mM of phosphate buffer (pH 7.0), 0.2 mL enzyme extract, 1 mL of 1 M H_2_O_2_, and 0.3 mL distilled water was used to test the CAT activity by calculating the reduction in absorbance measured at 240 nm within 3 min. One unit of CAT was determined by the amount decomposing 1 µmol of H_2_O_2_ within 1 min. 0.1 g leaves were extracted with 10 mL of 10% trichloroacetic acid (TCA) and then centrifuged at 10,000×g for 20 min.

Ascorbate peroxidase (APX) activity was measured by the method proposed by Yoshiyuki Nakano [79]. A total volume of 1 mL reaction mixture containing phosphate buffer (pH 7.0), 0.83 mL AsA, 0.13 mL H_2_O_2_, and 0.04 mL crude enzyme was utilized. The reduction in absorbance measured at 290 nm over 1 min was considered as the AsA consumption. The extinction coefficient of 2.8 mmol L^− 1^ cm was used to calculate APX activity. 0.1 g leaves were extracted with 10 mL of 10% trichloroacetic acid (TCA) and then centrifuged at 10,000 × g for 20 min.

### Statistical analysis

The values of each sample were processed using SPSS 20.0 (SPSS Inc., Chicago, IL, USA), based on one-way analysis of variance (ANOVA) with Duncan’s tests (p < 0.05). Origin (version 2021 Pro, Massachusetts, USA) was used for graphical representations. The correlations between the leaf length of the longest leaf, the leaf width of the longest leaf, the leaf number, the leaf fresh weight, the leaf dry weight, the leaf area were determined by Pearson test in Origin (version 2021 Pro, Massachusetts, USA).

## Data Availability

The datasets used and/or analysed during the current study are available from the corresponding author on reasonable request.
